# Genetic isolation in an endemic African habitat specialist

**DOI:** 10.1111/ibi.12520

**Published:** 2017-09-08

**Authors:** Natalie dos Remedios, Clemens Küpper, Tamás Székely, Neil Baker, Wilferd Versfeld, Patricia L. M. Lee

**Affiliations:** ^1^ Department of Animal and Plant Sciences University of Sheffield Western Bank Sheffield S10 2TN UK; ^2^ Milner Centre for Evolution Department of Biology and Biochemistry University of Bath Claverton Down Bath BA2 7AY UK; ^3^ Institute of Zoology University of Graz Universitätsplatz 2 8010 Graz Austria; ^4^ Max‐Planck‐Institute for Ornithology Eberhard‐Gwinner‐Str. 82319 Seewiesen Germany; ^5^ Tanzania Bird Atlas PO Box 1605 Iringa Tanzania; ^6^ Windpoort Farm PO Box 22 Okaukuejo Namibia; ^7^ School of Life and Environmental Sciences Centre for Integrative Ecology Deakin University Warrnambool Vic. 3280 Australia

**Keywords:** dispersal, phylogeography, population genetics, shorebirds, speciation

## Abstract

The Chestnut‐banded Plover *Charadrius pallidus* is a Near‐Threatened shorebird species endemic to mainland Africa. We examined levels of genetic differentiation between its two morphologically and geographically distinct subspecies, *C. p. pallidus* in southern Africa (population size 11 000–16 000) and *C. p. venustus* in eastern Africa (population size 6500). In contrast to other plover species that maintain genetic connectivity over thousands of kilometres across continental Africa, we found profound genetic differences between remote sampling sites. Phylogenetic network analysis based on four nuclear and two mitochondrial gene regions, and population genetic structure analyses based on 11 microsatellite loci, indicated strong genetic divergence, with 2.36% mitochondrial sequence divergence between individuals sampled in Namibia (southern Africa) and those of Kenya and Tanzania (eastern Africa). This distinction between southern and eastern African populations was also supported by highly distinct genetic clusters based on microsatellite markers (global *F*_*ST*_ = 0.309, $G_{ST}^{\prime }$ = 0.510, *D* = 0.182). Behavioural factors that may promote genetic differentiation in this species include habitat specialization, monogamous mating behaviour and sedentariness. Reliance on an extremely small number of saline lakes for breeding and limited dispersal between populations are likely to promote reproductive and genetic isolation between eastern and southern Africa. We suggest that the two Chestnut‐banded Plover subspecies may warrant elevation to full species status. To assess this distinction fully, additional sample collection will be needed, with analysis of genetic and phenotypic traits from across the species’ entire breeding range.

Genetic differentiation most commonly emerges between populations divided as a result of geographical barriers. Over evolutionary time, this process can result in the formation of distinct species from a single ancestral population via allopatric speciation (Mayr 1942, Avise 2009). In the absence of geographical barriers, and particularly in highly mobile organisms such as birds, populations can remain connected by dispersal over thousands of kilometres, thereby remaining genetically homogeneous across their continental range (Clobert *et al*. 2004, Claramunt *et al*. 2012).

The plovers (*Charadrius*) are shorebirds and include several species that exhibit high gene flow on a continental scale. For example, the Kentish Plover *Charadrius alexandrinus* of Eurasia remains genetically homogeneous across 10 000 km (Küpper *et al*. 2012) and Kittlitz's Plovers *Charadrius pecuarius* exhibit high levels of genetic homogeneity both across continental Africa (7600 km; dos Remedios 2013) and within Madagascar (Eberhart‐Phillips *et al*. 2015). For these species, genetic differentiation occurs only between populations separated by large bodies of water, such as between oceanic island and mainland populations (dos Remedios 2013, Almalki *et al*. 2017).

The Chestnut‐banded Plover *Charadrius pallidus* is distributed exclusively in continental Africa. The most recent taxonomic analyses suggest the species is a member of the Kentish Plover superspecies complex (dos Remedios *et al*. 2015). Yet despite its mainland distribution, two phenotypically differentiated subspecies are currently recognized. The taxon *C. p. pallidus* is distributed patchily in southern Africa, breeding in both coastal and inland wetlands in Namibia, Botswana and South Africa, with a fluctuating population of 11 000–16 000 individuals (Delany *et al*. 2009). In contrast, *C. p. venustus* is a non‐coastal species that inhabits a small number of saline lakes in the eastern African Rift Valley (Kenya and Tanzania), and includes approximately 6500 individuals (Simmons *et al*. 2007, Delany *et al*. 2009, Fig. 1a and 1b). In *C. p. pallidus*, individuals are approximately 15% larger, with paler and greyer upperparts than *C. p. venustus* (Hayman *et al*. 1986, Fig. 1a). However, whether these phenotypic differences are matched by genetic differentiation is unclear. *Charadrius* plovers are typically highly mobile and often exhibit considerable gene flow over large geographical distances (Funk *et al*. 2007, Küpper *et al*. 2012, D'Urban Jackson *et al*. 2017). Patterns of phenotypic and genetic differentiation do not necessarily match, making subspecies and species delineation in this taxonomic group often challenging (Rheindt *et al*. 2011, Küpper & dos Remedios in press).

**Figure 1:**
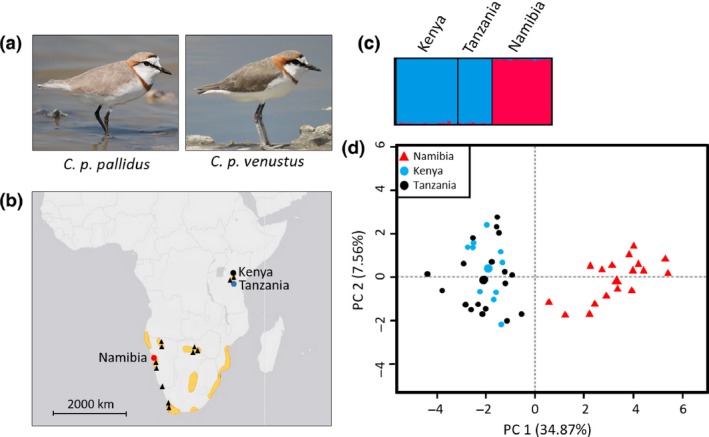
(a) Plumage coloration of breeding males in the southern African subspecies *Charadrius pallidus pallidus* and eastern African subspecies *Charadrius pallidus venustus*. (b) Distribution of Chestnut‐banded Plovers in Africa (triangles = sites holding 100 birds at least once, Simmons *et al*. 2007, dark shading = all recorded sightings, BirdLife International & Handbook of the Birds of the World 2016); labelled circles indicate sampling locations in southern Africa (Namibia; *C. p. pallidus*;* n* = 17 for microsatellite analyses) and eastern Africa (Kenya and Tanzania; *C. p. venustus*;* n* = 18 and 10, respectively, for microsatellite analyses). (c) Genetic clustering based on analysis of microsatellite loci using structure (*K* = 2) and (d) genetic clustering based on principal component analysis (larger symbols represent mean per cluster). Photo credits: Warwick Tarboton (*C. p. pallidus*) and Nik Borrow (*C. p. venustus*). [Colour figure can be viewed at http://onlinelibrary.wiley.com/journal/10.1111/(ISSN)1474-919X]

The Chestnut‐banded Plover has previously been described as being ‘overlooked’ by the scientific community (Simmons *et al*. 2007). It is considered Near Threatened (BirdLife International 2016) due to its reliance on an extremely small number of saline wetland sites during both the breeding and the non‐breeding seasons. In this study, we analyse nuclear and mitochondrial sequence data (six loci), as well as microsatellite markers (11 loci), to assess levels of genetic differentiation between the two subspecies of Chestnut‐banded Plover for the first time. We assess the validity of the current taxonomic classification and discuss the evolutionary history of this species.

## Methods

### Sample collection and DNA extraction

We sampled Chestnut‐banded Plovers at three locations (Fig. 1b): Mile 4 saltworks in Namibia (22°39′S, 14°33′E; subspecies *C. p. pallidus*), Lake Magadi in Kenya (1°52′S, 36°17′E; subspecies *C. p. venustus*) and Lake Manyara in Tanzania (3°40′S, 35°50′E; subspecies *C. p. venustus*). The distance between the Kenyan and Tanzanian sampling sites was approximately 150 km, whereas the distance between Namibian and Kenyan/Tanzanian sampling sites was more than 3000 km.

We captured individuals using mist‐nets or funnel traps following standard procedures (Székely *et al*. 2008). For blood sampling, we punctured the brachial wing vein with a hypodermic needle and used a capillary tube to transfer 25–50 *μ*L of blood to an Eppendorf tube for storage in 1 mL Queen's Lysis Buffer (Székely *et al*. 2008).

We selected putatively unrelated individuals, excluding known parent–offspring and sibling pairs, resulting in 73 samples for genetic analyses (21 from Namibia, 40 from Kenya and 12 from Tanzania). To extract DNA from these samples, we used an ammonium acetate precipitation method (Nicholls *et al*. 2000).

### Microsatellite analyses

We utilized microsatellite primers developed for the Kentish Plover (Küpper *et al*. 2007) and initially tested 18 markers in eight individuals. Four loci (Calex‐1, Calex‐13, Calex‐17, Calex‐34) were excluded from further analyses because of their low variability. We selected 14 polymorphic loci for which at least two different alleles were detected and designed two multiplexes using multiplex manager v1.2 (Holleley & Geerts 2009).

We conducted multiplex PCRs for all study individuals on a DNA Engine Tetrad 2 Peltier Thermal Cycler (Bio‐Rad, Hercules, CA, USA) in 2‐*μ*L reactions, including 10–20 ng DNA, 1 *μ*L Qiagen Multiplex PCR MasterMix and 1 *μ*L primers diluted in ddH_2_O (final primer concentration 0.2 mM; forward primers labelled with fluorescent dye), sealed with mineral oil to prevent evaporation. We carried out PCR amplification under the following conditions: 95 °C for 15 min, 35 cycles of 94 °C for 30 s, 56 °C for 90 s, 72 °C for 60 s, and finally 60 °C for 30 min. We visualized amplicons on an ABI 3730 automated DNA analyser and scored fragment lengths using genemapper software version 4.1 (Applied Biosystems, Waltham, MA, USA). Full genotype data are included in Table S1.

We ran 10% of samples twice (four samples from Kenya and two each from Namibia and Tanzania) to check consistency in allele scoring. Scoring was fully consistent across markers. We assessed heterozygosity in each population using cervus v3.0.3 (Kalinowski *et al*. 2007). Subsequently, we excluded three more loci (Calex‐8, Calex‐11, Calex‐23) from further analysis due to a high frequency of null alleles (> 0.2) and deviation from Hardy–Weinberg equilibrium across populations, leaving 11 loci in the final marker set (Table 1).

**Table 1:** Genetic variation and allele sizes for autosomal microsatellite loci among Chestnut‐banded Plovers

Locus	*n*	*A*	*H*_*o*_	*H*_*e*_	*F*_*ST*_	$G_{ST}^{\prime }$	*D*	Allele range	Multiplex	Dye
Calex‐02	44	3	0.34	0.52	0.137	0.242	0.131	150–154	B	NED
Calex‐04	45	4	0.40	0.63	0.486	0.772	0.554	207–215	A	NED
Calex‐07	45	5	0.44	0.58	0.119	0.223	0.136	149–163	A	6FAM
Calex‐16	44	2	0.18	0.17	0.010	0.006	0.001	207–209	B	NED
Calex‐18	45	4	0.38	0.46	0.035	0.078	0.038	162–168	A	NED
Calex‐19	45	4	0.13	0.54	0.787	0.923	0.624	298–304	A	NED
Calex‐33	45	5	0.42	0.64	0.402	0.683	0.482	153–161	A	HEX
Calex‐35	44	2	0.21	0.43	0.409	0.590	0.281	125–127	B	HEX
Calex‐39	45	5	0.64	0.73	0.034	0.122	0.090	121–135	A	HEX
Calex‐43	45	4	0.53	0.63	0.331	0.588	0.411	380–386	A	6FAM
Calex‐45	45	5	0.49	0.70	0.270	0.570	0.425	260–274	A	HEX
Mean of all loci	45	4	0.38	0.55	0.275	0.436	0.288			

*n*, number of individuals; *A*, number of alleles; *H*_*o*_, observed heterozygosity; *H*_*e*_, expected heterozygosity calculated based on the equation of Nei (1987). Fixation indices $G_{ST}^{\prime }$ and *D* are based on Hedrick (2005) and Jost (2008), respectively. Mean fixation indices are unweighted averages across loci; global fixation indices (weighted to account for unequal sample sizes) are reported in the text. For microsatellite primer sequences see Küpper *et al*. (2007).

Although sampled individuals were putatively unrelated based on field observations, evaluation of microsatellite data in ML‐relate (Kalinowski *et al*. 2006) suggested high levels of genetic relatedness *r* between some samples. We therefore excluded those with *r* > 0.5 from further analyses, leaving a sample size of 45 individuals (17 from Namibia, 18 from Kenya and 10 from Tanzania).

We estimated global fixation indices (*F*_*ST*_, $G_{ST}^{\prime }$ and *D*; Weir & Cockerham 1984, Hedrick 2005, Jost 2008) using the ‘diveRsity’ package in r (‘diffCalc’ function; Keenan *et al*. 2013) and inbreeding coefficients (*F*_*IS*_, i.e. deficit of heterozygosity relative to a randomly mating population; Weir & Cockerham 1984) using fstat 2.9.3 (Goudet 1995). We conducted analysis of molecular variance (amova) and assessed pairwise fixation indices (*F*_*ST*_) using arlequin 3.5.2 (Excoffier & Lischer 2010) to identify levels of genetic variance among sampling sites and subspecies.

Lastly, we used two methods to assess and visualize genetic structure between populations based on microsatellite data: principal component analysis (PCA; ‘princomp’ package in r (v3.0.3; R Core Team 2013)) and a Bayesian clustering method implemented in the software structure 2.3.4 (Hubisz *et al*. 2009). The latter was implemented with allele frequencies correlated for 500 000 Markov chain Monte Carlo repeats, after a burn‐in of 100 000, using an admixture model. We used structure harvester (Earl & vonHoldt 2012) to evaluate results, and produced plots with clumpp (Jakobsson & Rosenberg 2007) and distruct (Rosenberg 2004).

### Sequence analyses

We targeted six genes for sequencing, including two mitochondrial genes: COI (cytochrome oxidase I; Hebert *et al*. 2004) and ND3 (NADH dehydrogenase subunit 3; Chesser 1999), and four nuclear loci: ADH5 (alcohol dehydrogenase 5; Fain *et al*. 2007), FGB7 (*β*‐fibrinogen intron 7; Prychitko & Moore 1997)*,* MB2 (Myosin‐2/3; Slade *et al*. 1993) and RAG1 (recombination activating gene 1; Groth & Barrowclough 1999).

We conducted PCRs for three individuals from each sample site. Reactions of 10 *μ*L total volume contained 4 *μ*L Qiagen Multiplex Mix, 0.1 *μ*M of each primer and 20–30 ng DNA, and were PCR‐amplified on a DNA Engine Tetrad 2 Peltier Thermal Cycler. PCR conditions were as follows: 95 °C for 15 min, followed by 42 cycles of 94 °C for 30 s, *T*_*a*_ (58 °C for COI and FGB7; 62 °C for ND3 and MB2; 64.5 °C for ADH5 and RAG1) for 30 s, 72 °C for 30 s and a final extension of 72 °C for 10 min. We tested a 3‐*μ*L aliquot of the PCR products on a 1.5% agarose gel to determine amplification success. We purified PCR products using 2 *μ*L 10× diluted ExoSAP‐IT (GE Healthcare Chicago, IL, USA) according to the instructions of the manufacturer. Cycle‐sequencing was performed by GenePool Laboratory, Edinburgh, UK, on an ABI 3730 DNA analyser (Applied Biosystems) using the BigDye Terminator v3.1 cycle sequencing kit (Applied Biosystems).

Sequences were attained for all nine individuals across all loci. We aligned manually edited sequences in codoncode aligner 3.7.1 (CodonCode Corporation, Centerville, MA, USA) using the ClustalW algorithm. We submitted sequence data to GenBank (for accession numbers see Table 2 and Table S1). We used DNasp v5 (Librado & Rozas 2009) to assess nucleotide variation across sampled populations. Lastly, we carried out phylogenetic network analyses using pofad software (Phylogeny of Organisms From Allelic Data; Joly & Bruneau 2006) based on three datasets, the first incorporating sequences from all six loci, the second including only mitochondrial loci (COI and ND3) and the third including only nuclear loci (ADH5, FGB7, MB2 and RAG1). Uncorrected pairwise genetic distances were initially calculated for each locus using mega6 (Tamura *et al*. 2013) and pofad was then used to generate a single unstandardized distance matrix for each of the three multi‐locus datasets. Networks were constructed using the Neighbor‐Net algorithm in splitstree4 v4.14.4 (Huson & Bryant 2006).

**Table 2:** GenBank accession numbers for sequence data generated from Chestnut‐banded Plovers

Sampling site	COI	ND3	ADH5	FGB7	MB2	RAG1
Kenya	KM001292	KM001385	KM001129	KM001464	KM001213	KM001551
	KM001293	KM001386	KM001130	KM001465	KM001214	KM001552
	KM001294	KM001387	KM001131	KM001466	KM001215	KM001553
Namibia	KX371163	KX371124	KX371202	KX371222	KX371179	KX371143
	KX371164	KX371125	KX371203	KX371223	KX371180	KX371144
	KX371165	KX371126	KX371204	KX371224	KX371181	KX371145
Tanzania	KX371166	KX371127	KX371205	KX371225	KX371182	KX371146
	KX371167	KX371128	KX371206	KX371226	KX371183	KX371147
	KX371168	KX371129	KX371207	KX371227	KX371184	KX371148

## Results

Our analyses of both microsatellite and sequence data indicated that the Chestnut‐banded Plovers of Kenya and Tanzania form a distinct genetic cluster separate from those of Namibia.

For microsatellite loci, measures of heterozygosity and allele ranges are included in Table 1. The mean number of alleles per locus was four (mean expected heterozygosity *H*_*e*_ 0.548). amova attributed 38.93% of molecular variance at microsatellite loci to differences between the Namibian individuals and those of Kenya/Tanzania, whereas there was no molecular variance detectable between Kenya and Tanzania (−0.41%, Table 3).

**Table 3:** Analysis of molecular variance (amova), based on 11 microsatellite loci for the best grouping of Chestnut‐banded Plovers: Namibia (*Charadius pallidus pallidus*) distinct from Kenya/Tanzania (*Charadius pallidus venustus*)

	df	*SS*	*Va*	%
Among groups (*C. p. pallidus* – *C. p. venustus*)	1	63.6	1.46	38.93
Among populations within groups	1	1.9	−0.02	−0.41
Within populations	87	200.6	2.31	61.48
Total	89	266.1	3.75	

df, degrees of freedom; *SS*, sum of squares; *Va*, variance; %, percentage of total variance. This grouping had the highest ‘among group’ variance based on separate amova analyses for each possible grouping of the three populations.

The results of structure analysis strongly supported the presence of two genetic clusters (Fig. 1c; *K* = 2, Delta *K* = 1622.6 vs. *K* = 3, Delta *K* = 20.6) corresponding to southern and eastern African populations. Individuals were assigned with high confidence to each cluster (membership coefficients > 0.955; mean 0.992). Similarly, these two genetic clusters were clearly identifiable based on PCA, with the first principal component (PC 1) explaining 34.87% of total variance and the second (PC 2) explaining 7.56% of variance at microsatellite loci (Fig. 1d).

Global fixation indices based on microsatellite loci indicated substantial genetic differentiation among populations (global *F*_*ST*_ = 0.309, $G_{ST}^{\prime }$ = 0.510, *D* = 0.182; see Table 1 for per‐locus indices). Pairwise fixation indices between sampling locations were largest for comparisons between southern and eastern African sampling sites (Kenya – Namibia: *F*_*ST*_ = 0.381, *P* < 0.001; Tanzania – Namibia: *F*_*ST*_ = 0.388, *P* < 0.001) but low and not different from zero for the within‐East African sample site comparison (Kenya – Tanzania: *F*_*ST*_ = −0.006, *P* = 0.766). Inbreeding coefficients *F*_*IS*_ for each location were: Namibia 0.078 (*P* = 0.115), Kenya 0.142 (*P* = 0.017), Tanzania 0.092 (*P* = 0.162).

Based on sequence data, fixed nucleotide substitutions were identified between eastern Africa (Kenya/Tanzania) and Namibia at mitochondrial loci (COI, 12 fixed substitutions across 615 sites; ND3, nine fixed substitutions across 398 sites), but not at nuclear loci (ADH5 693 sites, FGB7 817 sites, MB2 679 sites, RAG1 911 sites). In total, 3100 sites were analysed across four nuclear loci and just three substitutions were present among Tanzanian individuals. Average sequence divergence at mitochondrial loci was 2.36% (COI 1.95%, ND3 2.76%) compared with < 0.01% at nuclear loci.

**Figure 2:**
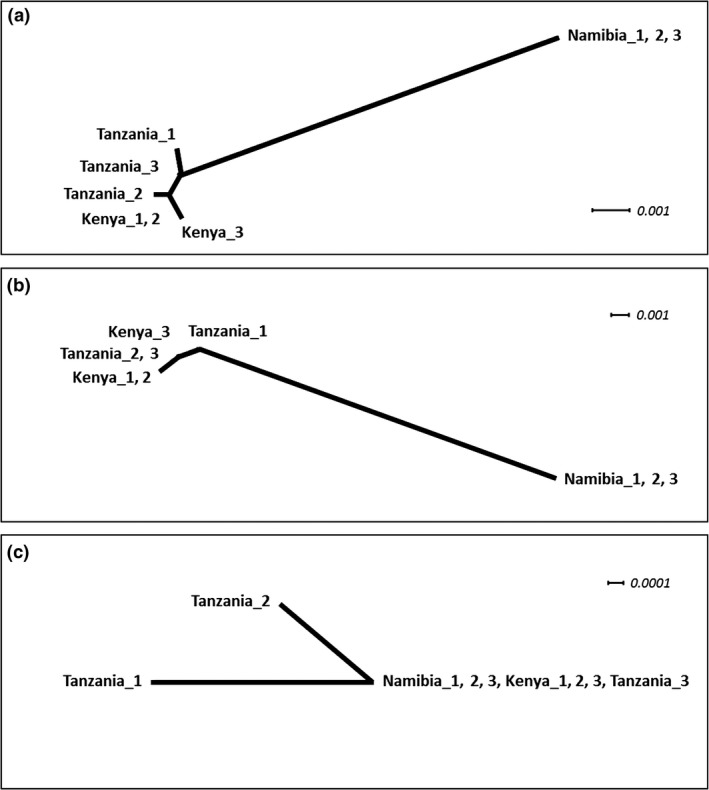
Phylogenetic networks based on multi‐locus distance matrices for sequence data (generated using pofad), constructed with a Neighbor‐Net algorithm (splitstree4 v4.14.4) for (a) nuclear and mitochondrial loci combined, (b) mitochondrial loci (cytochrome oxidase I, COI; NADH dehydrogenase subunit 3 genes, ND3) and (c) nuclear loci (alcohol dehydrogenase 5, ADH5; *β*‐fibrinogen intron 7, FGB7; Myosin‐2/3, MB2; recombination activating gene 1, RAG1). Across loci, three individuals (labelled 1, 2 and 3) were sequenced from each location (Kenya, Namibia and Tanzania). Scale bars indicate genetic distance (substitutions per site).

Results of phylogenetic network analyses based on mitochondrial sequence data supported assignment of Namibian individuals to a distinct genetic cluster from those of Kenya and Tanzania (Fig. 2). This clustering was also reflected in the combined mitochondrial and nuclear analysis. However, extremely low sequence variation at nuclear loci led to limited genetic clustering based on nuclear sequences (Fig. 2), with Tanzanian individuals exhibiting the only polymorphism, as described above.

## Discussion

We identified strong genetic divergence between the two currently recognized subspecies of Chestnut‐banded Plover, *C. p. venustus* of eastern Africa and *C. p. pallidus* of southern Africa, based on microsatellite genotyping and mitochondrial sequence analyses. Genetic structure and estimated levels of inbreeding can be affected by including related individuals in the analysis (Rodríguez‐Ramilo & Wang 2012). To test the robustness of our results, we carried out all microsatellite analyses with both the full dataset and a conservative dataset taking into consideration the probability of relatedness. Results were qualitatively similar in both cases (full dataset included in Table S1), indicating that strong genetic differentiation is indeed present between the two subspecies of Chestnut‐banded Plover and is not an artefact of sample selection. In the conservative dataset, inbreeding coefficients (*F*_*IS*_) indicated a significant heterozygote deficit among Kenyan individuals, suggesting that levels of inbreeding may be higher at the Kenyan site in contrast to the Namibian and Tanzanian sites.

The classification of subspecies does not always directly correspond to genetic differences (Zink 2004). A recent study of 296 Scandinavian bird species reported greatly overlapping ranges of intraspecific and interspecific divergence (mean divergence in COI sequences was 0.24% within species and 7.95% between sister species; Johnsen *et al*. 2010). In the Chestnut‐banded Plover, we identified COI sequence divergence of 1.95% and overall divergence at mitochondrial genes (COI and ND3) of 2.36%. This is greater than the divergence between other closely related *Charadrius* species (dos Remedios 2013), the Kittlitz's Plover and St Helena Plover *Charadrius sanctaehelenae* (0.49% mitochondrial divergence), and similar to that between the Kittlitz's Plover and Madagascar Plover *Charadrius thoracicus* (2.54% mitochondrial divergence), but also comparable to subspecies‐level divergence among island and mainland populations of the Three‐banded Plover *Charadrius tricollaris* and White‐fronted Plover *Charadrius marginatus* (1.05 and 1.68%, respectively). In line with clear morphological differences, our analyses of microsatellite loci also confirmed that the two Chestnut‐banded Plover subspecies belong to distinct genetic clusters, with genetic differentiation between subspecies far greater than between populations of the same subspecies.

Based on these diagnostic genetic differences as well as the documented morphological differences (Hayman *et al*. 1986), elevation to full species status may be warranted for *C. p. pallidus* and *C. p. venustus*. Our current results indicate that dispersal of Chestnut‐banded Plovers between eastern and southern Africa is limited and, with continued isolation, these taxa are likely to diverge further. This strong genetic divergence is surprising given that for most other *Charadrius* species studied to date, dispersal and gene flow between populations occur over much greater continental distances (Küpper *et al*. 2012, Küpper & dos Remedios in press). For example, the Kittlitz's Plover exhibits high population connectivity and genetic homogeneity across Africa and its range overlaps that of the Chestnut‐banded Plover (D'Urban Jackson *et al*. 2017). We suggest that behavioural factors such as habitat specialization and mating behaviour may be responsible for maintaining isolation between Chestnut‐banded Plovers in southern and eastern Africa, and that unlike other *Charadrius* plovers, these behaviours may lead to non‐oceanic barriers restricting dispersal in this species.

The Chestnut‐banded Plover is a habitat specialist usually found within a short distance of saline or alkaline water (< 50 m during the breeding season, Hockey *et al*. 2005; < 1 km during the non‐breeding season, Simmons *et al*. 2007), with a preference for areas devoid of vegetation (Johnsgard 1981). Populations of *C. p*. *venustus* in eastern Africa are resident with a strongly localized distribution that reflects this habitat specialization. Records suggest that wanderings are confined to the eastern Rift Valley, with rare cases of breeding by vagrants in ephemeral wetlands no more than 300 km south of the breeding population at Lake Manyara. This is far short of the closest population of the nominate subspecies, 2000 km away in northern Botswana (Nata delta; Simmons *et al*. 2007). Individuals of *C. p. pallidus* from Namibia, Botswana and South Africa exhibit greater movement and may be resident, nomadic or migratory (Delany *et al*. 2009). Yet, although non‐breeding vagrants have been recorded as far north as coastal Mozambique, south of the Zambezi River (Hockey *et al*. 2005), no suitable breeding habitat has been recorded in Mozambique, Malawi, Zambia or Zimbabwe (N. Baker unpubl. data). The lack of genetic admixture found in our study suggests that dispersal is unlikely to enable mixing with eastern African *C. p*. *venustus* populations, although breeding populations of *C. p. pallidus* from the east coast of South Africa should to be sampled to confirm this.

Mating behaviour may also facilitate genetic differentiation relative to some other plover species. The Chestnut‐banded Plover is monogamous (Hockey *et al*. 2005) and therefore likely to exhibit a high degree of philopatry (Saalfeld & Lanctot 2015, D'Urban Jackson *et al*. 2017). This behaviour is in contrast to the behaviour of sequentially polygamous plovers, in which one parent deserts their brood shortly after hatching to remate, sometimes in a new location, leaving their partner to provide care for the offspring (Küpper *et al*. 2012). Mating behaviour and associated breeding dispersal may have a large impact on genetic differentiation. Overall, greater genetic structure has been identified among monogamous than polygamous plover populations (D'Urban Jackson *et al*. 2017) and this may contribute to the lack of genetic mixing between populations of the Chestnut‐banded Plover.

With a behavioural tendency towards sedentariness, geographical isolation is likely to decrease population connectivity over a smaller scale than for more widely dispersing species. Subspecies *C. p. venustus* avoids both freshwater and coastal areas such that today, the inland saline wetlands of the East African Rift Valley offer the only suitable breeding habitat for 2000 km between central Tanzania and northern Botswana (N. Baker unpubl. data). In addition, these wetlands are bordered by some of the highest mountains in Africa, as well as some of the deepest freshwater lakes. To understand the evolutionary history of Chestnut‐banded Plover populations and how they reached their current state of isolation, it is important to consider the influence of long‐term climatic and geographical changes.

Although it is not possible to date the divergence of the two subspecies without further molecular clock analyses, studies in a range of African bird species have highlighted the importance of palaeoclimatic events and habitat changes on patterns of dispersal, vicariance and speciation (Fjeldså & Bowie 2008, Voelker & Light 2011, Oatley *et al*. 2012, Voelker *et al*. 2012, 2014, Kahindo *et al*. 2017). Open savannah and grassland habitats have dominated much of sub‐Saharan Africa (with the exception of the Guinea–Congolian forests) for the last few million years (Voelker *et al*. 2012). During this time, dramatic landscape changes have occurred in eastern Africa. First, habitats here have been affected by long‐term aridification with episodic periods of extreme humidity (Maslin *et al*. 2014, Voelker *et al*. 2014, Kahindo *et al*. 2017). Secondly, mountain ranges and deep lake basins continued to form as the central plateau of Africa uplifted. This resulted in the formation of the modern rift escarpments of the Magadi–Natron basin within the last 2 million years (Foster *et al*. 1997, Trauth *et al*. 2005), now home to *C. p. venustus*. In contrast, south‐western Africa, home to *C. p. pallidus*, has remained relatively stable both geologically and tectonically for the last 3.5 million years, providing refugia for many taxa (Maslin *et al*. 2012). It seems inevitable that these factors would have influenced the evolutionary history of Chestnut‐banded Plovers and their present‐day distribution.

The elevation of subspecies *C. p. venustus* and *C. p. pallidus* to full species should result in a further review of the conservation status of each taxon. Chestnut‐banded Plovers are currently considered to be Near Threatened (BirdLife International 2016) and are especially vulnerable as they depend on an extremely small number of sites. As many as 87% of individuals are reported to congregate in just three locations during the non‐breeding season (Walvis Bay and Sandwich Harbour in Namibia, and Lake Natron in Tanzania; Simmons *et al*. 2007) with only eight sites holding more than 1% of the population. Our results confirm that Chestnut‐banded Plovers disperse only short distances and therefore may not be capable of re‐locating elsewhere should any of these sites become untenable. They would therefore be highly vulnerable in the face of global threats to wetland habitat (Davidson 2014, Dixon *et al*. 2016). The inclusion of key sites in the Ramsar List of Wetlands of International Importance (Walvis Bay, Sandwich Harbour and Lake Natron) should aid the protection of the species in these locations, yet without full protection of the surrounding areas, continued conservation efforts will be needed to maintain the quality of habitats in these biologically diverse regions.

Before reclassifying the two subspecies of Chestnut‐banded Plover as distinct species, we recommend the collection and analysis of genetic samples from across the full southern African range of *C. p. pallidus*, including breeding populations not covered by our work. Augmenting our current genetic analyses with more broad‐scale sequencing at additional loci would not only clarify the taxonomic status of these populations but also allow full delineation of geographical distributions and the estimation of divergence times in their evolutionary history. Additionally, we suggest the examination of morphological data, detailing phenotypic variation in plumage, body size, ecology, life history and behaviour across the range of the species. Regardless of their taxonomic status, the genetic differentiation identified between *C. p. pallidus* and *C. p. venustus* indicates that each should be managed independently as a distinct evolutionary unit rather than a combined Africa‐wide population.

We thank Nelli Rönkä, Rauri Bowie and two anonymous reviewers for their valuable comments on drafts of the manuscript. For collection of samples, we thank Bernard Amakobe, Sylvester Karimi, Christine Ochieng and the National Museums of Kenya. Sequence analysis was supported by NERC‐Biomolecular Analysis Facility at the University of Edinburgh and at the University of Sheffield (NBAFE/S547). This study was funded by the NERC as part of the PhD research of N.dR. C.K. was supported by a Marie Curie IEF Postdoctoral Fellowship. Permits for fieldwork and collection of samples were provided by the National Council for Science and Technology, Kenya, the Namibian government, Ministry of Environment and Tourism, and the Tanzania Commission for Science and Technology (COSTECH) and Tanzania Wildlife Research Institute (TAWIRI).

## Supporting information


**Table S1**. Microsatellite genotype data for all individuals (14 loci; *n* = 73) and GenBank accession numbers for sequence data (six loci; *n* = 9).
